# Probing O(^3^P) Reactivity with Chemisorbed Hydrocarbons: Insights from Experiment and Theory

**DOI:** 10.1021/acs.jpca.5c01709

**Published:** 2025-06-03

**Authors:** Claudia Bennett-Caso, Angelina L. Leonardi, Rachel Hambuchen, Aida Castelblanco, Jack Spagnoletti, Cecily Szady, Natasha Wozniak, Juan G. Navea

**Affiliations:** Chemistry Department, 7230 Skidmore College, Saratoga Springs New York 12866-1632, United States

## Abstract

Heterogeneous oxidation of hydrocarbons via low-pressure nonthermal plasma has traditionally focused on nonvolatile compounds since volatile or semivolatile hydrocarbons can partition into the plasma, reducing product selectivity. However, adsorption of volatile compounds can prevent the hydrocarbon from vaporizing and reaching the plasma-phase, allowing reactions to take place between free radicals generated via nonthermal plasmas and the volatile or semivolatile hydrocarbon. In this work, we present a state-of-the-art chamber for the heterogeneous reactions between adsorbed hydrocarbons and ground-state oxygen (O­(^3^ P)) generated via nonthermal plasma. The chamber enabled a hydrocarbon monolayer on an alumina thin film to be exposed to a plasma plume generated with a radio frequency (RF) generator. In situ vibrational spectroscopy of the alumina-coated surface was used to investigate the relative kinetics of two model hydrocarbons, 1-hexene and cyclohexane, chemisorbed onto alumina. Finally, the functionalization of the chemisorbed hydrocarbon on the alumina powder via nonthermal plasma was investigated in situ in order to determine the conditions for an effective oxidation. Our results show a novel and effective method for the reaction with adsorbed volatile compounds with O­(^3^P). For the adsorbed compounds, the reaction of adsorbed cyclohexane is twice as fast as adsorbed 1-hexene, which represents a significant change with respect to gaseous phase rates, where 1-hexene reaction with O­(^3^P) is approximately 37 times faster than cyclohexane.

## Graphical abstract

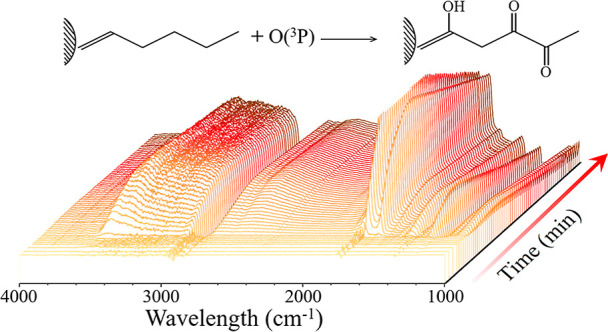

## 1 Introduction

Efficiently and selectively functionalizing volatile and semivolatile organic compounds remains a significant challenge in the chemical and petrochemical industries due to the relative stability of their C–H and C–C bonds, which resist most transformation methods.^1–5^ In particular, functionalization methods that introduce oxygen-containing functional groups to alkanes and alkenes have been proposed to enhance fuel quality, improve engine performance, and reduce exhaust emissions.^6–10^ Traditional hydrocarbon oxidation methods often rely on high-temperature heterogeneous catalysis,^11^ which can be disadvantageous when applied to volatile and semivolatile fractions of petroleum-derived compounds, often causing gas-phase partitioning, fractionation, reduced selectivity, and overoxidation.^1^ In addition, the high-temperatures required for these heterogeneous oxidation processes are energetically demanding.^1,2,12^ Another approach to oxidizing hydrocarbons and other relatively inert compounds involves treatment with ozone, which can be effective but demands careful operation due to potential safety risks.^13–15^ While enzymatic oxidation has been used for the functionalization of alkanes and alkenes, these methods can often be impractical due to the high cost and the relatively slow reaction rates.^16,17^ Overall, these challenges highlight the need for a method that can achieve selective transformations of inert hydrocarbons without the high temperatures typical of cracking and other similar processes, which can transform the more volatile or semivolatile fractions from petroleum-derived compounds.

Over the past two decades, nonthermal (or nonequilibrium) plasmas have been proposed as a viable method for generating ground-state atomic oxygen, O­(^3^P), to drive the heterogeneous oxidation of hydrocarbons, producing oxygenated products such as ketones, epoxides, and alcohols. Nonthermal plasmas offer the key advantage of enabling oxidation reactions at low temperatures, which minimizes the side reactions common at higher temperatures and allows for relatively selective hydrocarbon oxidation. However, selectivity is maintained as long as the hydrocarbons remain in a condensed phase; once volatilized, the hydrocarbons undergo ionization in the plasma plume, leading to fractionation and multiple reaction pathways typical of homogeneous plasma processes. Recent work has focused on the partial oxidation of long-chain alkanes using a nonthermal, atmospheric-pressure, oxygen-rich plasma.^18,19^ Using long-chain alkanes like *n*-octadecane, a solid at room temperature, enabled oxidation to proceed without volatile organic emissions, resulting in relatively high selectivity.^20^ Similarly, prior studies on plasma oxidation have typically employed larger, nonvolatile alkanes under low-pressure, oxygen cold-plasma conditions to produce O­(^3^P) at ambient temperatures.^21^ In contrast, low-pressure oxygen cold plasmas have also been applied to smaller-chain hydrocarbons (C6 to C10). Patiño and co-workers were among the first to use this approach, using a cryogenic bath to prevent the liquid hydrocarbon from entering the plasma phase.^22–24^ More recently, condensed propene films at cryogenic surface temperatures have been used to study O­(^3^P) reactions with propane.^25^ While these methods effectively generated oxidized products without the typical high-temperature conditions, generating selectively secondary alcohols and ketones, the reactions were largely constrained to hydrocarbons with low volatility at the application temperature.

The need to cool the reaction in a cryogenic bath, which allowed the reaction to proceed without hydrocarbon volatilization, limited the ability to perform a detailed kinetic study and prevented a molecular-level description of the interfacial process between the liquid and plasma plume containing O­(^3^P). As a result, in situ reactions of hydrocarbons with low-pressure plasmas remain largely unexplored. In this study, we investigate the use of a thin film surface to facilitate heterogeneous reactions between a low-pressure nonthermal plasma and chemisorbed hydrocarbons. Here, hydrocarbons remain in a condensed phase by chemisorbing onto an alumina surface site, forming a monolayer over the available surface. Alumina, commonly found in mineral dust,^26–29^ fly ashes^30–33^ —byproducts of coal combustion—, and other combustion residuals,^34^ presents a cost-effective and readily available surface for hydrocarbon functionalization. Through combined spectroscopic and computational approaches, we analyze the molecular-level interactions between chemisorbed 1-hexene and cyclohexane—two volatile hydrocarbons—and a low-pressure plasma. Through chemisorption, these hydrocarbons remain in a condensed phase, preventing volatilization and enabling the heterogeneous reaction to proceed at the low pressure required for nonthermal plasmas. This molecular-level study, which includes a kinetic analysis, highlights key variations in the reactivity of the two isomers of C_6_H_12_ at an interface exposed to oxygen plasma compared with known values in the gas phase.

## 2 Experimental Section

### 2.1 Plasma Generation and Reaction Chamber

A custom-built reaction chamber, a stainless-steel cube of 100.45 cm^3^ internal volume, coupled with a nonthermal plasma system, was designed for in situ analysis of surface-bound hydrocarbon oxidation by O­(^3^P) (Figure 1). Nonthermal O­(^3^P) plasma was generated using a commercially available 13.4 MHz radio frequency (RF) generator (Manitou Systems) applied over a low-pressure of extra dry O_2_ flow (>99.9% purity). This frequency was selected to prevent interference with external communications and electronic systems. The setup generates a nonthermal plasma beam within a set of concentric discharge glass tubes, centrally positioned inside a copper coil. The inner tube of the concentric system extends inside the reaction chamber positioned slightly off-center toward one side of the chamber. The outer tube is secured to the top flange of the reactor chamber by using an Ultratorr adaptor, ensuring system pressure stability, as illustrated in Figure 1B. The reaction chamber is connected to a vacuum line at the bottom, positioned opposite the plasma discharge tube, as shown in Figure 1A. This configuration ensures that the plasma beam travels across the chamber from the entry point to the opposite side. The vacuum line, equipped with traps to capture volatile products and protect the mechanical vacuum pump, provides overall control of the pressure inside the chamber with the flow of the O_2_ adjusted by a regulator and pressure monitored using a digital pressure gauge in the vacuum line.

**Figure 1:**
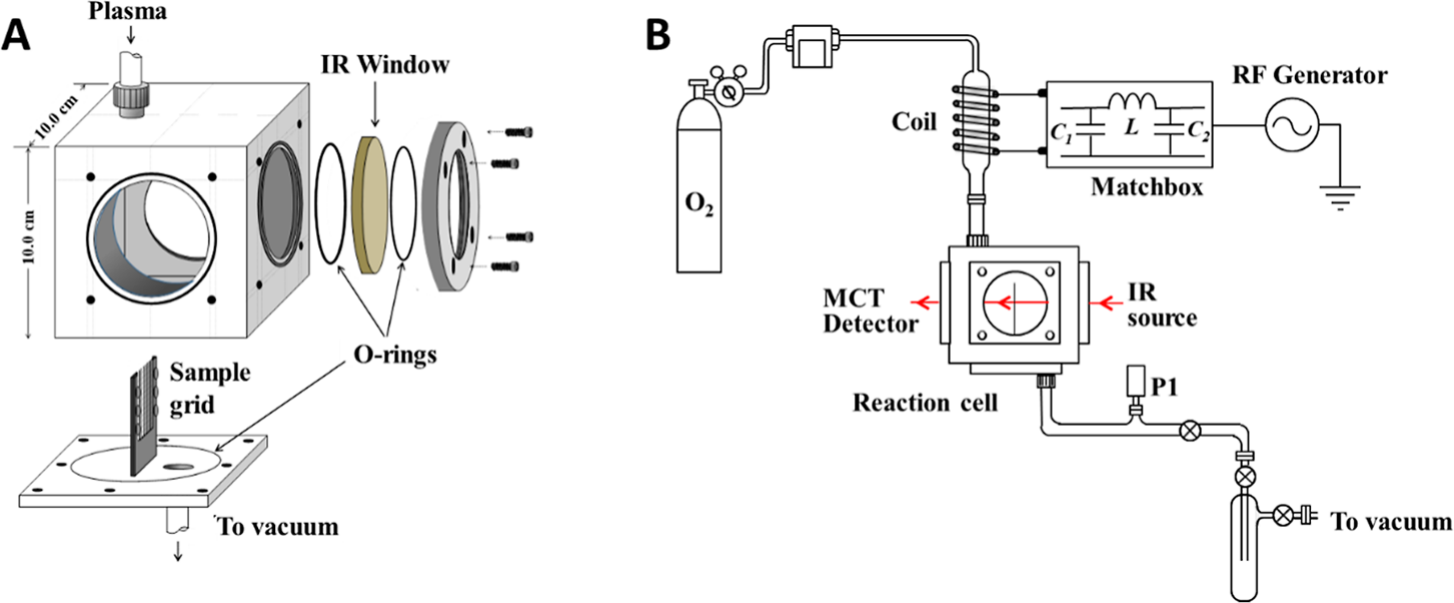
Schematic diagram of the vibrational spectroscopy flow system. The experimental apparatus consists of (A) a vacuum-sealed chamber enclosing the tungsten grid that holds the sample. The chamber is equipped with four sets of perpendicular windows, BaF_2_ IR windows for vibrational spectroscopy and quartz windows for emission UV–vis spectroscopy. The chamber is connected to a (B) flow low pressure system, equipped with a radio frequency (RF) generator for the generation of oxygen nonthermal plasma.

While the top and bottom faces of the reaction chamber are used for the plasma discharge tube and a vacuum port, the lateral faces are equipped with windows for in situ spectroscopy. Perpendicular to the plasma beam direction, barium fluoride windows allow for vibrational spectroscopy analysis, while quartz windows, aligned parallel to the plasma beam path, facilitate UV–vis emission analysis.^35^ At the center of the chamber, a tungsten grid (32 × 32 wires per cm, 0.01 cm wire diameter) is positioned perpendicular to the plasma beam, forming a 90° angle with its direction. This grid supports a thin film of alumina coated with the hydrocarbon of interest, either 1-hexene or cyclohexane. Vibrational spectroscopy has been successfully used by our group and others to study interfaces, proving to be a valuable tool under varying pressures, temperatures, and other environmental conditions.^36–39^ In the system presented here, the plasma plume directly contacts the upper third of the coated tungsten grid, aligning with the point where the infrared (IR) beam intersects the sample for transmission FTIR spectroscopy (Figure 2A). This design enables vibrational spectroscopic analysis of the chemisorbed species as they interact with O­(^3^P) in the nonthermal plasma. Finally, the reaction chamber is placed in a sample compartment of a Fourier transformed infrared (FTIR) spectrophotometer (Thermo, iS50), equipped with a liquid nitrogen cooled narrow band mercury cadmium telluride (MCT) detector for rapid spectral acquisition. A commercial air dryer (Parker) was used to purge the FTIR interferometer and the sample compartment around the reaction chamber in order to minimize interferences from atmospheric H_2_O and CO_2_.

**Figure 2:**
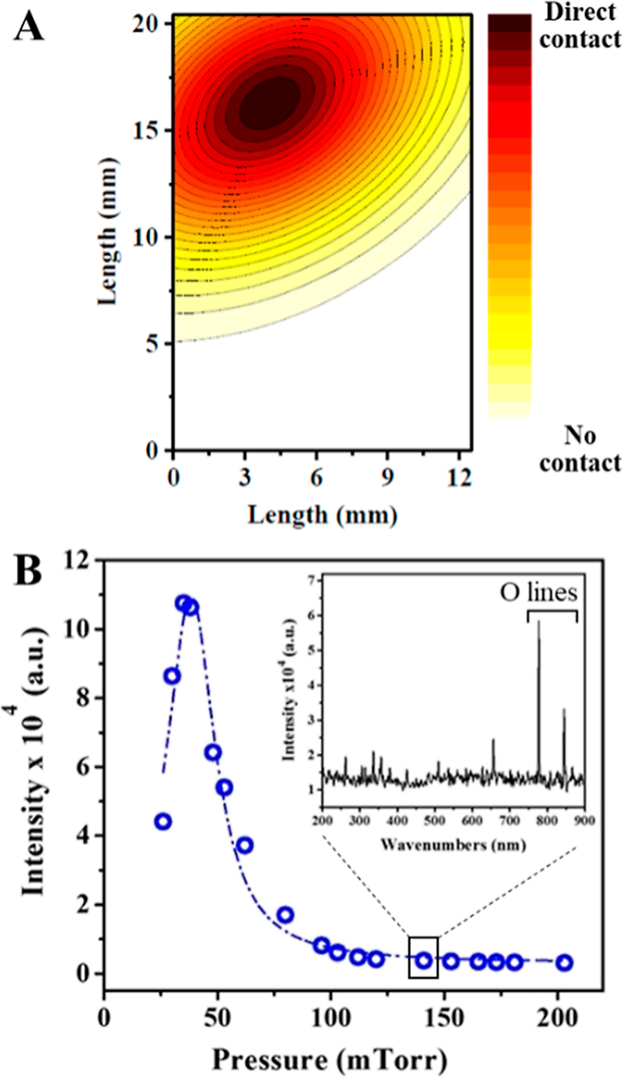
(A) Mapped image of a catechol-coated α-Al_2_O_3_ film after oxidation with an O­(^3^P) nonthermal plasma plume; the color mapping indicates the relative intensity of oxidation based on the integrated areas of the carbonyl area band, with the darker colors showing larger oxidation features. (B) Optical emission intensity of the oxygen plasma as a function of pressure. Insert: UV–vis spectral emission at 140 mTorr, showing the lines at 777 and 844 nm, corresponding to transitions O­(3p^5^ P → 3s^5^S) and O­(3p^3^ P → 3s^3^S), respectively.

In a typical experiment, a slurry of alumina powder (α-Al_2_O_3_), with a surface area of (99.2 ± 0.5 m^2^ g^–1^), suspended in either cyclohexane or 1-hexene (C_6_H_12_) is deposited onto the tungsten grid so that the alumina film coating the grid is saturated with the hydrocarbon. The thin film, coated with either 1-hexene or cyclohexane, is positioned at the center of the reaction chamber for transmission FTIR measurements, with the infrared beam interacting with the thin film in the same position as the plasma beam. Before experimentation, the system is evacuated to approximately 40 mTorr for several minutes to remove any gas-phase or physisorbed hydrocarbons. Initially, α-Al_2_O_3_ coated with the C_6_H_12_ isomer likely consists of both chemisorbed and physiosorbed molecules, with chemisorption limited to a monolayer. Beyond the monolayer, adsorption enthalpies approach the enthalpy of vaporization under standard conditions, meaning the vapor pressure of the physiosorbed compound is similar to its standard value.^40,41^ Since the vapor pressures of 1-hexene (155 Torr) and cyclohexane (99 Torr) at the reaction temperature (22 °C) far exceed the system pressure (40 mTorr), physiosorbed molecules are expected to desorb, leaving only a monolayer or submonolayer coverage of chemisorbed C_6_H_12_.

After the removal of physiosorbed compounds, the system is brought to the desired pressure of 140 mTorr, with a continuous O_2_ flow, ensuring that only a monolayer of chemisorbed C_6_H_12_ remains on the α-Al_2_O_3_ thin film. The pressure of 140 mTorr of O_2_ is sufficient to form an O­(^3^P) plasma beam, enabling the oxidation reaction

1 C6H12(a)+O(P3)→Products

where C_6_H_12_(*a*) represents either chemisorbed cyclohexane or 1-hexene. The formation of a minimum amount of O­(^3^P), approximately 0.045̅ times the maximum formation of O­(^3^P), ensures a sufficiently low reaction rate, facilitating time-resolved monitoring of the reaction. No ozone formation was detected during the generation of the plasma.

Typically, a background is collected consisting of the thin film with the chemisorbed hydrocarbon under an O_2_ flow at the working plasma pressure. Thus, all transmission FTIR absorbance spectra collected every 22 s, consisting of 50 scans with a resolution of 4 cm^–1^, are referenced to the adsorbed C_6_H_12_ so that positive features represent the formation of functional groups related to products, while valleys represent the removal of a reactant functional group. Ex situ analysis is performed by collecting the α-Al_2_O_3_ thin film with all surface-bound species. The coated powder is immersed in acetone for solvent extraction, followed by a passivation period lasting several days. The acetone is then analyzed using gas chromatography–mass spectrometry (GC–MS) to identify and quantify surface-bound products. No volatile products were observed in the liquid nitrogen traps during the experiments.

### 2.2 Nonthermal Plasma Characterization and Optimization

The oxygen atom plume was characterized using emission atomic spectroscopy, while FTIR microscopy of the tungsten grid—still coated with a postreaction alumina thin film—was used to determine the area of the plasma’s interaction with the sample. Here, catechol was chemisorbed onto a α-Al_2_O_3_ thin-film to map the plasma contact and oxidation of the surface-bound sample as the oxidation of catechol produces *o*-benzoquinone, identifiable by its intense carbonyl bands. The plasma–sample contact area was mapped by exposing the thin film to a 75 mTorr oxygen nonthermal plasma for 5 min. FTIR microscopic analysis of the carbonyl bands after oxidation revealed the intensity of oxidation across the surface, indicating the plasma plume’s point of contact (Figure 2A). The plasma plume collides with the grid slightly off the vertical centerline, forming a Gaussian distribution concentrated at the top of the grid. No detectable oxidation was observed near the bottom of the grid. The infrared beam used for in situ transmission FTIR, as described in Section 2.1, intersects the grid in the region near high plasma contact, crossing at the top and at the vertical centerline.

Nonthermal plasmas require low-pressure conditions, where the carrier gas pressure significantly influences plasma behavior. High intermolecular interactions and the low-energy requirement for plasma generation limit the stability of the plasma plume. To determine the optimal pressure for O­(^3^P) atoms generation, oxygen pressure in the reaction chamber was systematically varied, and the oxygen atoms’ emission intensity was monitored. The plasma emission was obtained using a high-resolution UV–vis spectrometer equipped with fiber optics (Ocean Optics), positioned perpendicular to the IR beam at the quartz window (Figure 1), with the emission focused onto the fiber optic by using fused silica lenses. The primary emission lines observed at all working pressures that generated a discharge were at 777.6 and 844.7 nm, corresponding to transitions O­(3p^5^P → 3s^5^S) and O­(3p^3^P → 3s^3^S), respectively, as shown in the inset of Figure 2B.^24,42^ These lines were consistently observed under all experimental conditions, where O_2_ predominantly dissociates into ground-state O­(^3^P) atoms.^1,22,44^ While metastable excited-state species, such as O­(^1^D), may be present in the plasma, they undergo rapid quenching through collisions with other plasma species, with a quenching lifetime of approximately 5.5 μs (see Supporting Information), causing them to relax to the more stable ground-state O­(^3^P).^1,22,23,45–47^ As a result, particularly in nearly pure O_2_ dissociations, the density of O­(^3^P) is significantly higher in the plasma beam.^45^ The absence of the 557 nm emission line, associated with the transition of O­(^1^S) → O­(^1^D), suggests that the density of O­(^1^S) is too low to be detected. Thus, despite the potential presence of excited states, the plasma beam is predominantly composed of O­(^3^P), making it the primary reactive species.

Although the findings indicated a peak plasma intensity at approximately 50 ± 5 mTorr, operational conditions were set at 140 mTorr to ensure a reaction rate—dependent on the flux of O­(^3^P)—sufficiently slow for conducting comparative kinetic analysis. The steep slope of the intensity curve on either side of the peak at 50 mTorr suggested that even minor fluctuations in pressure could result in significant variations in the concentration of the O­(^3^P) and plasma reactivity. By maintaining a pressure of 140 mTorr, a more stable and consistent intensity of O­(^3^P) was achieved, thus maximizing reproducibility and ensuring reliable oxidation reactions of surface-bound hydrocarbons. This optimization enhances the overall reproducibility of the experimental conditions.

### 2.3 Quantum Chemical Calculations of Chemisorbed C_6_H_12_

To facilitate the interpretation of the vibrational spectra and provide some insight into the geometric aspects of chemisorbed 1-hexene and cyclohexane, electronic structure optimizations and vibrational frequency calculations were conducted using Gaussian 09 quantum chemical software, with the results visualized using GaussView software. Quantum chemical computations focused on binuclear clusters, which simulated a geometrically constrained Al_2_O_3_ surface site, with either 1-hexene or cyclohexane placed in various initial configurations from the surface site.^48^ Energy minimizations were then performed on each cluster using Becke’s three-parameter hybrid method with the LYP correlation functional (B3LYP). In this study, the ground-state structures of C_6_H_12_ adsorbed on an Al_2_O_3_ cluster ([(Al­(OH)_3_)_2_(C_6_H_12_)]) were optimized using the B3LYP/6-311G­(d) basis set, which was found to be suitable for geometry optimization based on comparisons with experimental data.^49^ With terminal hydrogens excluded, the structures were optimized by freezing the aluminum oxide surface site, yielding C_6_H_12_ coordinations that represent the global minima of the potential energy surface and the favored chemisorption configurations.^26,48^ Vibrational frequency calculations, used to interpret the loss of frequencies as the reaction progresses, were also performed at the B3LYP/6-311G­(d) level of theory. To account for anharmonicity, all calculated frequencies were scaled by a factor of 0.9737.^50–52^ The C–H stretching modes (ν_CH_) exhibit a larger anharmonicity compared to other vibrational bands of lower energy in the Mid-IR.^53,54^ To accurately reproduce experimental results, we incorporate this anharmonicity, further red-shifting the ν_CH_ bands using an empirical scaling factor of 0.9273,^55^ which improves agreement with the experimental spectra.^53,55^ All calculations assume no intermolecular interactions between adsorbates as only one site and one C_6_H_12_ molecule were calculated.

## 3 Results and Discussion

### 3.1 Theoretical Model of the Adsorbed Hydrocarbon

The optimized geometry of 1-hexene and cyclohexane chemisorbed onto the model surface site of alumina (Al­(OH)_3_)_2_)^48^ is shown in Figure 3A and B, respectively. The global minimum for adsorbed 1-hexene on alumina corresponds to a configuration where the double bond is near the surface, allowing the π electron density to help minimize the energy of the Al_2_O_3_–C_6_H_12_ binuclear cluster. This suggests that the sp^3^ carbons are positioned away from the surface, leaving them more exposed to an Eley–Rideal-type reaction, in which adsorbed 1-hexene reacts with the nonthermal plasma flux. Additionally, adsorption restricts the degrees of freedom of the 1-hexene motion, keeping the molecule largely in its global minimum configuration. As a result, the double bond in adsorbed 1-hexene may be less accessible for the interaction with plasma species than those in liquid hydrocarbons exposed to nonthermal plasmas. When 1-hexene is adsorbed on an alumina surface, its bond length undergoes minimal changes. The double bond length decreases slightly from 1.32981 Å in the gas phase to 1.32525 Å (Figure 3A). Similarly, the C–H bond lengths in the sp^2^ carbons decrease by less than 0.8% compared to their gas-phase values. The most significant change in adsorbed 1-hexene compared to its gaseous phase geometry is in the alkene dihedral angles, which shift from 0.28185° (for the two C–H in the cis position) and 0.65986° (for C–C_4_H_9_ and C–H) in the gas phase to 2.65812° and 1.78997°, respectively. The increase in the alkene dihedral angle causes a slight reorientation of the saturated chain in 1-hexene, shifting it away from the surface site and increasing the steric hindrance around the double bond.

**Figure 3:**
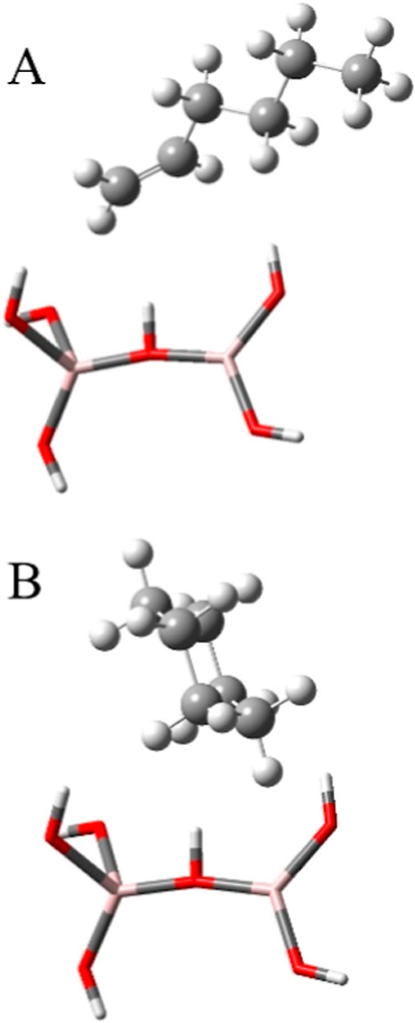
Global energy minima of (A) 1-hexene and (B) cyclohexane adsorbed onto the alumina active site optimized at the B3LYP/6-311G­(d) level of theory. The hydrocarbon isomers C_6_H_12_ are shown with a ball and stick model, and the surface site is shown with a tube frame. Clusters presented here correspond to energy minima matched with experimental vibrational frequencies.

Adsorbed cyclohexane, shown in Figure 3B, undergoes a loss of symmetry upon adsorption, shifting from its D_3d_ symmetry in the chair configuration to a slightly distorted, yet stable, chair form. In this adsorbed state, the carbon near the surface site bends slightly, reducing the adjacent C–C–C bond angles from 111.5°—a near-ideal tetrahedral geometry with minimal angle strain—to 110.6°. This distortion lowers the molecule’s symmetry from D_3d_ to C_s_. In this geometry, one end of the molecule interacts with the surface, while the other half remains exposed and available for interactions with nonthermal plasma species. Although adsorbed cyclohexane has restricted motion compared to that in liquid or gaseous phase, its higher symmetry—unlike 1-hexene—is largely preserved upon adsorption, as the angular distortion is relatively small. This retained symmetry allows its collisions with O­(^3^P) from the nonthermal plasma flume to still resemble those in the liquid phase.

### 3.2 Nonthermal Plasma Oxidation

The time-dependent transmission FTIR spectra of 1-hexene and cyclohexane chemisorbed on Al_2_O_3_ during their reaction with O­(^3^P) are shown in Figure 4A and B, respectively. Each spectrum is referenced to the adsorbed hydrocarbon on the Al_2_O_3_ surface. Initially, under an O_2_ pressure of 140 mTorr before the RF is on, no oxidation of the chemisorbed hydrocarbons is observed, indicated by the unchanged vibrational spectra shown in the baseline before plasma ignition (darkest colors in Figure 4). At a time *t* = 0, the RF discharge is initiated, igniting the nonthermal plasma and producing a flux of O­(^3^P). This marks the onset of chemisorbed C_6_H_12_ oxidation, as evidenced by distinct features in the IR spectra. The thin films of chemisorbed hydrocarbon show both a significant increase and a decrease in absorption features relative to the O­(^3^P) exposure time. In Figure 4, spectra corresponding to longer reaction times are shown as lighter colors. Positive features, or bands above the baseline, correspond to vibrational bands of reaction products, while negative bands, shown as valleys below the baseline, indicate the vibrational bands from the depleted functional groups of the adsorbed hydrocarbon, as it undergoes reaction and it is consumed. Compared to cyclohexane, 1-hexene exhibits a higher infrared absorbance in the vibrational spectra of its reaction products with O­(^3^P), which we interpret as a higher surface affinity for Al_2_O_3_ due to its π-bonds and geometry, as shown in Figure 3A, allowing for a higher surface density of 1-hexene. This greater surface density enhances spectral sensitivity, enabling better resolution at longer reaction times due to the concomitant stronger absorbance of its oxidation products.

**Figure 4:**
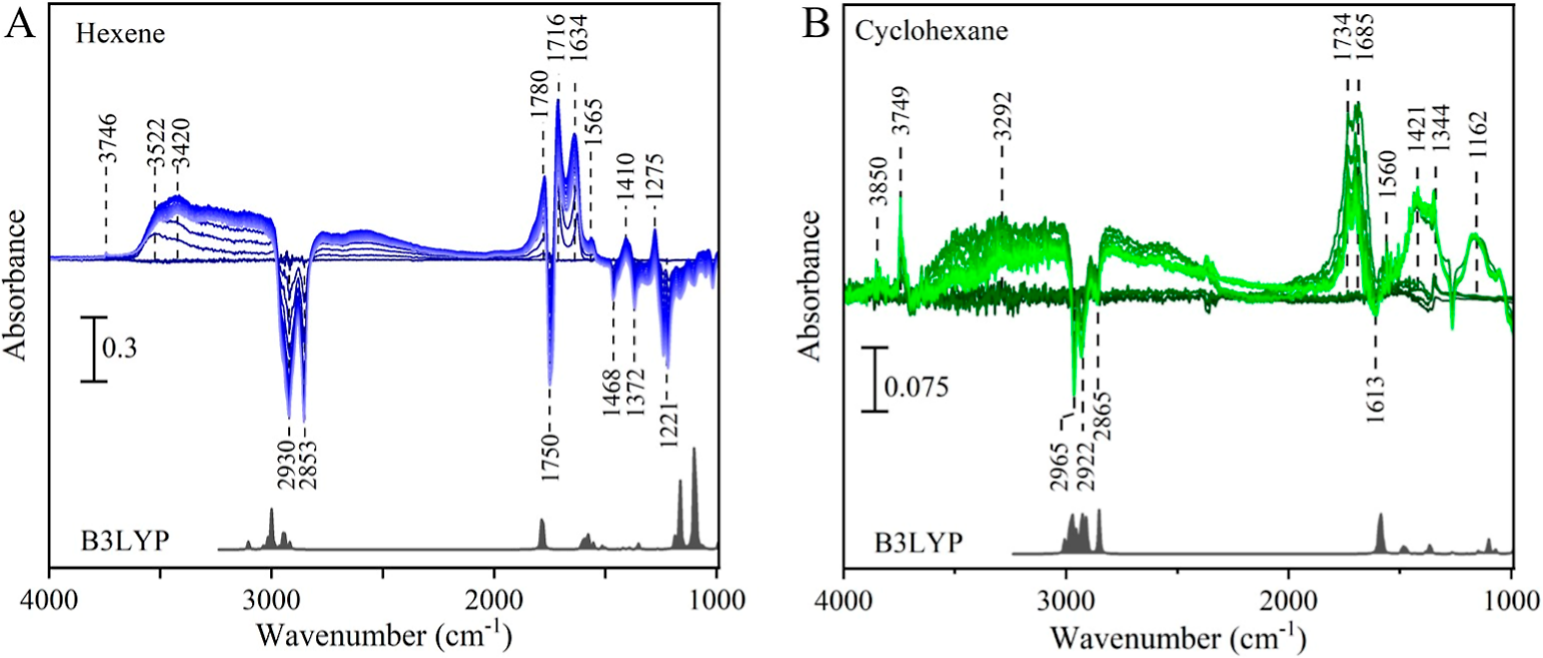
Time-resolved FT-IR spectra of O­(^3^P) reactions with hydrocarbons adsorbed on Al_2_O_3_. (A) 1-Hexene over 30 min and (B) cyclohexane over 15 min. All spectra are referenced to the adsorbed hydrocarbon. Absorbance intensities reflect the initial density coverage, with 1-hexene exhibiting higher density due to its adsorption geometry. The spectra are presented at 220 s intervals, with lighter lines indicating later times. The gray spectra at the bottom of each panel shows the calculated vibrational spectra adjusted for anharmonicity.

Upon exposure to O­(^3^P), both chemisorbed 1-hexene and cyclohexane show a significant increase in absorption intensity in the 3000 to 3600 cm^–1^ region, a broad band assigned to the stretching (ν_OH_) modes of alcohol functional groups, indicating the formation of alcohol-containing oxygenated products in both C_6_H_12_ isomers. Figure 4 shows the initial growth of the ν_OH_ bands, followed by a decline in their intensity at longer reaction times. This is indicated by the lighter-colored spectra, which represent extended O­(^3^P) exposure, not coinciding with the maximum absorption intensity of the aqueous O­(^3^P) to the aqueous O­(^3^P) at an earlier time in the reaction. The formation of alcoholic functional group reaches a maximum at around 15 min for adsorbed 1-hexene, while for adsorbed cyclohexane, the maxima of this absorption band is observed at just 2 min of reaction time. In Figure 4A, the vibrational spectra for the reaction of O­(^3^P) with adsorbed 1-hexene show a small but observable band centered around 3746 cm^–1^. For the reaction of O­(^3^P) with adsorbed cyclohexane, more intense sharp absorption bands centered at 3749 cm^–1^ and 3850 cm^–1^ are also observed to grow with reaction time. Contrary to the bands between 3000 and 3600 cm^–1^, this sharp ν_OH_ does reach a maximum at longer O­(^3^P) exposure times, as evidenced by the lighted color corresponding to the more intense absorption. These bands are attributed to isolated hydroxyl group terminals on the surface of α-Al_2_O_3_, which start to grow as the coverage changes, leaving some surface sites available.^33,35,56^ Continued exposure to the nonthermal plasma plume results in a continued oxidation of the organic coverage, leading to a maximum in the sharp ν_OH_ absorption bands. This is evident in the bottom panels of Figure 5, which present a heatmap of vibrational spectra over time. The sudden formation of a broad band between 3000 and 3600 cm^–1^, characteristic of organic alcohols, appears upon plasma ignition, reaches a maximum, and then decreases in intensity. In contrast, the sharp band above 3700 cm^–1^ continues to increase with the reaction time. The plasma ignition is clearly observed in Figure 5, indicated in the left axis of the heatmap, but clearly distinguishable in the intensity of the vibrational bands associated with the oxygenated functional groups.

**Figure 5:**
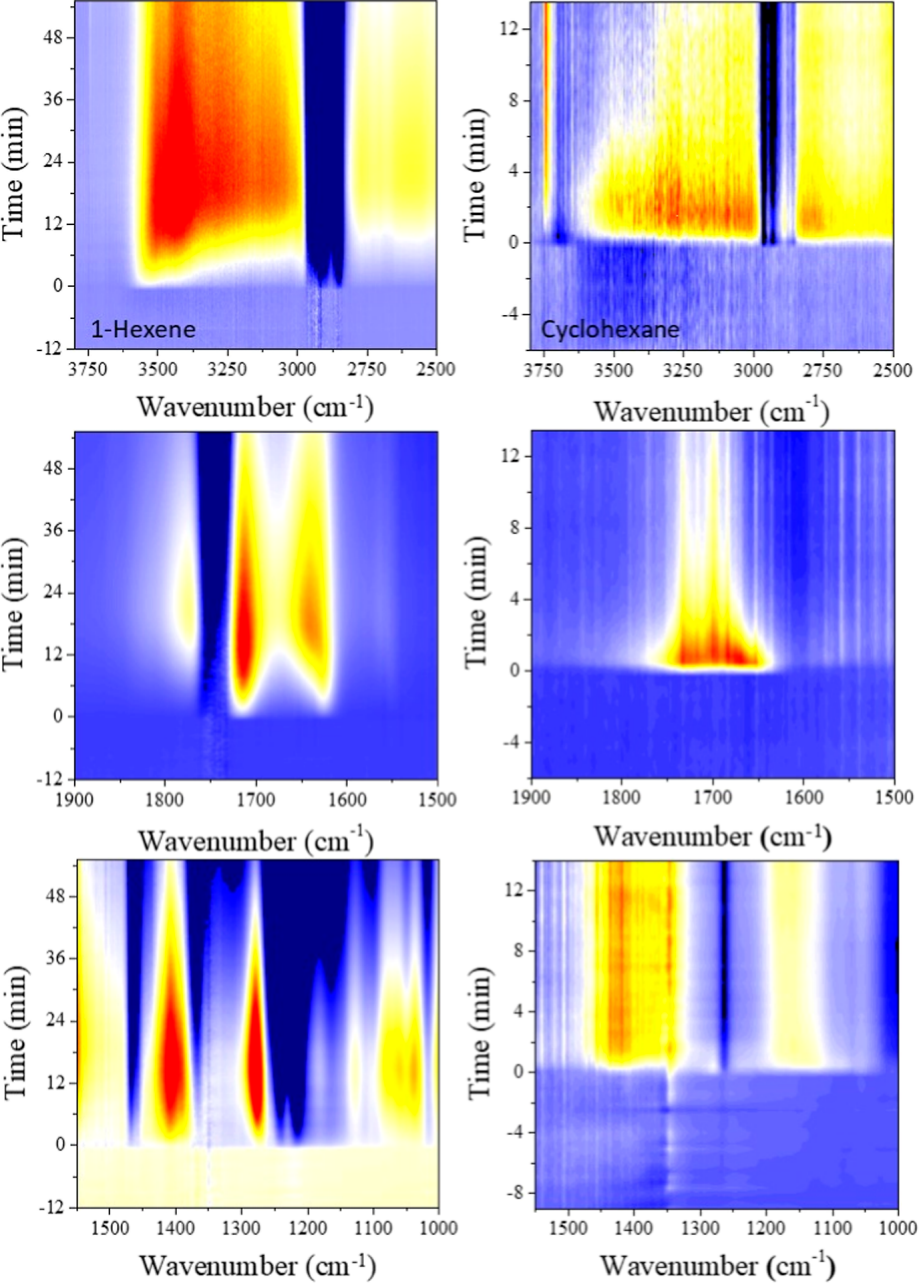
Vibrational spectral data of 1-hexene (left column) and cyclohexane (right column) in reaction with O­(^3^P). Timescales for 1-hexene and cyclohexane reactions are different. Warmer colors indicate peak growth (yellow, orange, red), and cooler colors (blue) indicate peak degradation.

Figure 4 also shows that the reaction between O­(^3^P) and both chemisorbed 1-hexene and cyclohexane leads to the growth of positive absorptions bands at 1780 and 1680 cm^–1^ due to carbonyl C=O stretches from the formation of multiple ketone species, characteristic of oxidation products.^22–24,36^ Finally, the growth of the absorption bands at 1410 and 1275 cm^–1^ in Figure 4A and 1421 and 1344 cm^–1^ in Figure 4B can be assigned to the combination of C–H bending mode and O–H in-plane bending from ketone and alcohol products as well as a combination of C–O stretching vibrations from other oxygenated species.^22–24,36^ The negative absorption bands observed at 2930 and 2853 cm^–1^ in Figure 4A and at 2965, 2922, and 2865 cm^–1^ in Figure 4B arise from a decrease in the C–H stretching absorbance. This absorbance loss is due to the reaction of O­(^3^P), which initiates hydrogen atom abstraction from the chemisorbed hydrocarbon, as has been proposed when atomic oxygen, or similar species, reacts with an organic fraction (Scheme 1).^22–24,57–62^ For the reaction between O­(^3^P) and chemisorbed 1-hexene, two experimental ν_CH_ negative bands are observed to grow with the reaction time. The first, centered at around 2930 cm^–1^, is attributed to a combination of the C–H asymmetric stretch of the methyl group, the C–H asymmetric stretches of the chain sp^3^ carbons, and the asymmetric C–H stretch of the double bond. The second, a lower-energy ν_CH_ band centered at 2853 cm^–1^, corresponds to the C–H symmetric stretch of the sp^3^ carbon in 1-hexene. As shown in the gray theoretical spectra in Figure 4A, a less intense band at higher energy (∼3100 cm^–1^) is associated with the symmetric C–H stretch of the alkene hydrogens, though it is likely contained within the broader ν_CH_ at 2930 cm^–1^. For the reaction between O­(^3^P) and chemisorbed cyclohexane, there are broadly speaking three ν_CH_ band groups that show in both the theoretical spectra and the spectra in Figures 4 and 5. The less intense ν_CH_ band centered at 2865 cm^–1^ corresponds to the C–H stretch of carbon near the alumina surface. The vibrational band at 2922 cm^–1^ is attributed to a combination of symmetric stretches, while the more intense band at 2965 cm^–1^ is assigned to asymmetric C–H stretches farther from the surface site.

**Scheme 1:**
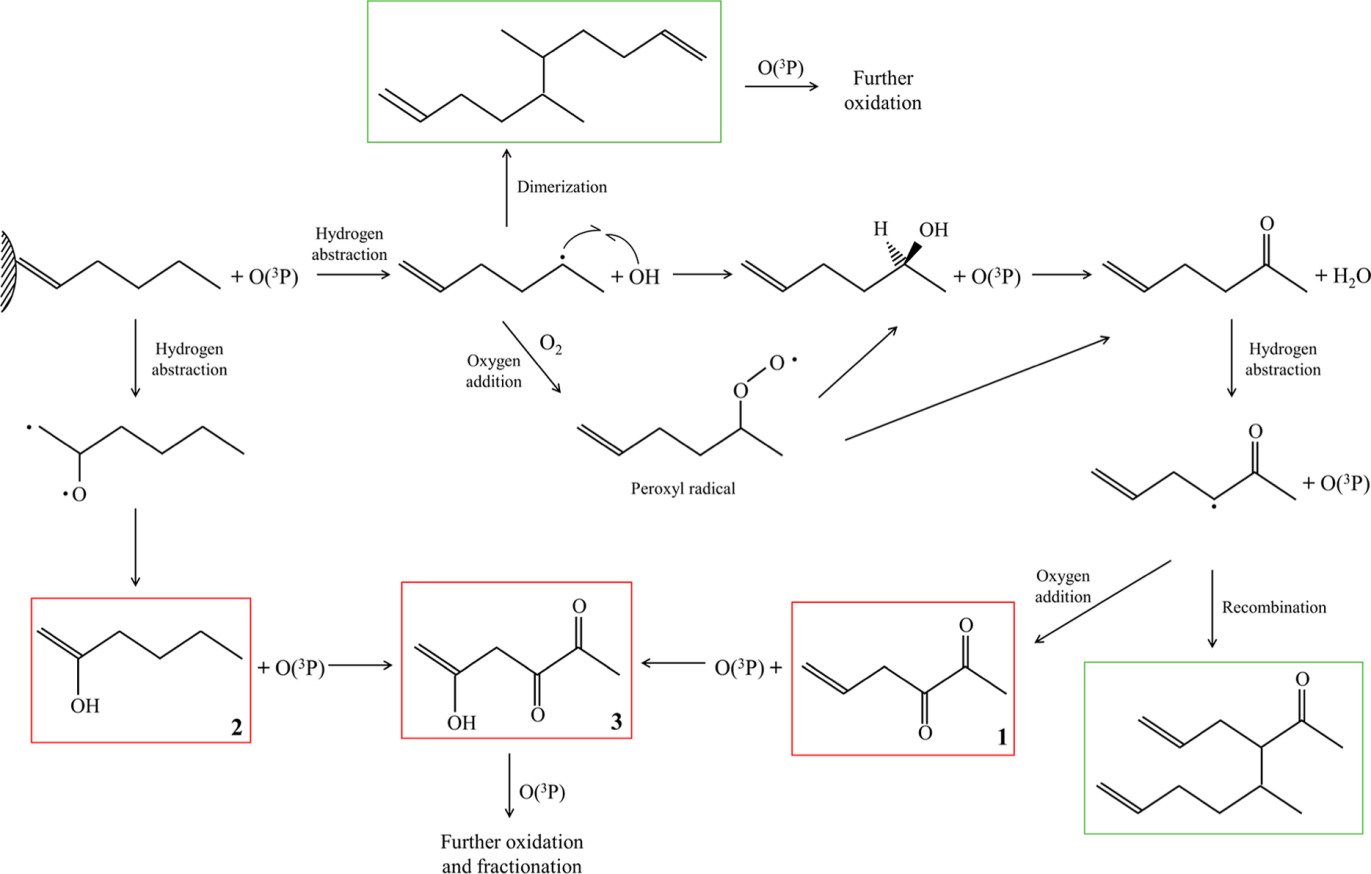
Nonthermal Oxygen Plasma Reaction with 1-Hexene Chemisorbed on α-Al_2_O_3_ ^a^

a All steps of the reaction involved surface bound hydrocarbons, including intermediaries and products. Red boxes show products detected via GC-MS. Green boxes show possible recombination products with masses >160 amu. Numbers indicate the products identified via GC-MS, as shown in Figure 6.

The positive absorption bands at 1780 and 1680 cm^–1^ observed in Figures 4A and 5, attributed to ketone formation, are truncated by a strong negative band centered at 1750 cm^–1^, which corresponds to the carbon–carbon double bond stretching. This is in good agreement with the theoretical spectra in Figure 4A, where the alkene stretch is centered at around 1780 cm^–1^. As adsorbed 1-hexene is exposed to O­(^3^P), it reacts to form ketones and alcohols, as described in Scheme 1.^57,58^ The negative bands around 1410 and 1275 cm^–1^ are attributed to the scissoring CH mode of adsorbed 1-hexene, which appears at approximately 1555 cm^–1^ in the theoretical spectra shown in the gray inset of Figure 4A. Similarly, the negative bands around 1221 cm^–1^ correspond to the in-plane (δ_CH_) and out-of-plane (γ_CH_) C–H bending modes of the alkene group in adsorbed 1-hexene, observed at 1165 cm^–1^ and 1100 cm^–1^ in the theoretical spectra, respectively. For the reaction of adsorbed cyclohexane with O­(^3^P), a valley at 1613 cm^–1^ corresponds to the C–H scissoring mode of cyclohexane, in good agreement with the theoretical spectra, where it appears at 1600 cm^–1^. Figure 5 shows that the increase in the intensity of the negative bands for both chemisorbed 1-hexene and cyclohexane remains consistent with reaction time and does not reverse direction with exposure to the O­(^3^P) plume, as in the case of the OH and carbonyl bands.

Postreaction GC–MS analysis of surface-bound products confirms the vibrational spectroscopy results. Figure 6 presents the characterization and quantification of oxygenated products from the Eley–Rideal-type reaction, where O­(^3^P) in the plasma plume reacts with surface-bound species.^63^ The oxygen addition reaction produces a combination of ketones and alcohols for both 1-hexene and cyclohexane,^64–66^ with some recombination and dimerization leading to higher-mass surface-bound products.^67^ Schemes 1 and 2 show the proposed mechanisms for adsorbed 1-hexene and cyclohexane, respectively. In both cases, the initial rate-determining step involves hydrogen abstraction, leading to alcohol formation.^64–66,68,69^ As oxidation progresses, ketone groups form. While some fractionation and volatilization of products may occur, potentially leading to compounds such as acetaldehyde and formaldehyde,^66^ the decrease in absorbance intensity of surface-bound products with O­(^3^P) exposure time (after reaching a maximum) does not result in the detection of volatile products.

**Figure 6:**
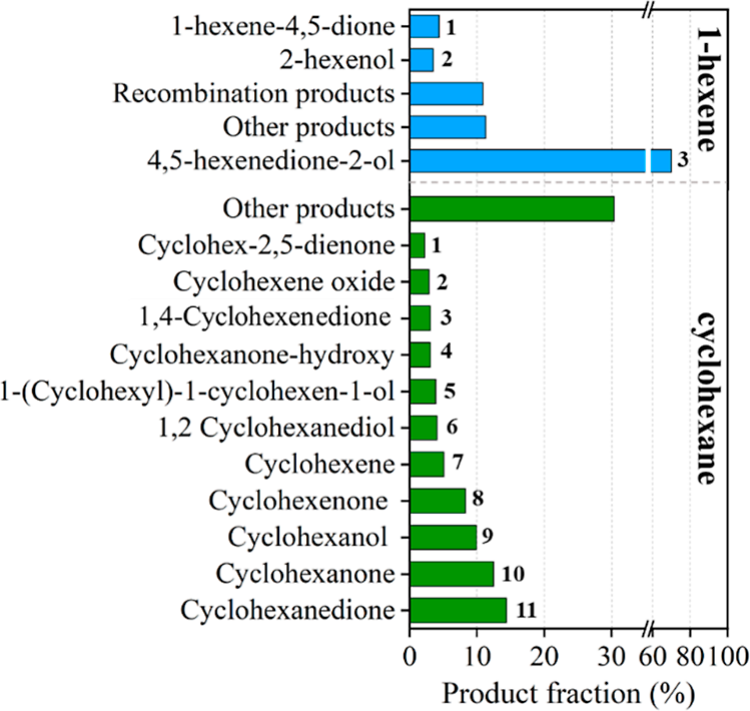
Postreaction GC–MS analysis of surface-bound products. The top (blue) part shows the main products of the chemisorbed 1-hexene reaction with O­(^3^P), and the bottom (green) part shows the main products of the chemisorbed cyclohexane reaction with O­(^3^P). Numbers correspond to the products in Schemes 1 and 2.

**Scheme 2:**
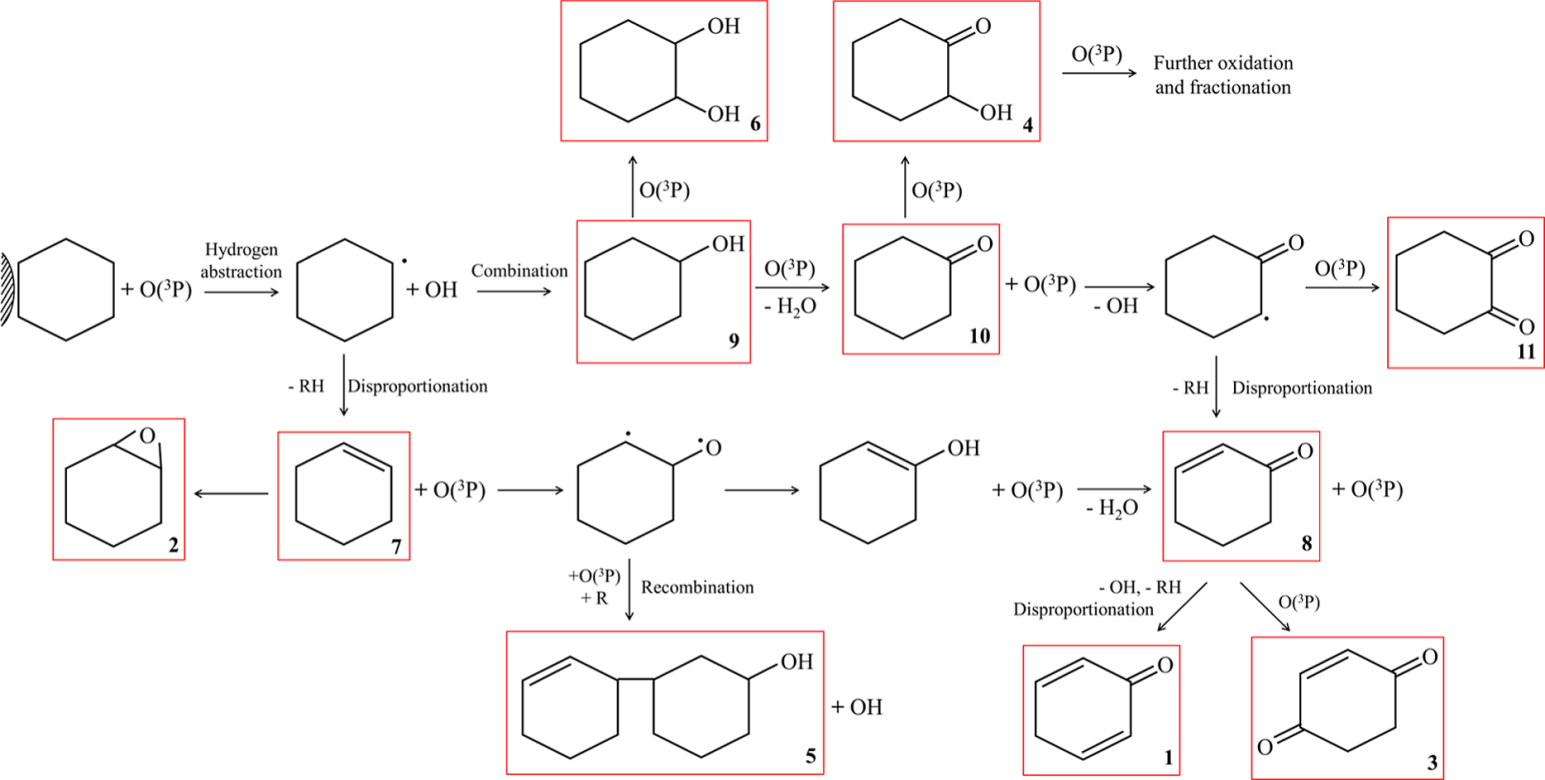
Nonthermal Oxygen Plasma Reaction with Cyclohexane Chemisorbed on α-Al_2_O_3_ ^a^

a All steps of the reaction involved surface-bound hydrocarbons, including intermediaries and products. Red boxes show products detected via GC-MS. Numbers indicate the products identified via GC-MS, as shown in Figure 6

Figure 6 suggests that the oxidation of adsorbed 1-hexene is more selective than the reaction between O­(^3^P) and adsorbed cyclohexane, yielding not only a narrower range of products but also predominantly forming 4,5-hexenedione-2-ol, which accounts for nearly 70% of the total products. Specifically, the formation of oxidation products at the sp^3^ carbon chain, rather than at the nucleophilic alkene group, indicates a surface effect in which the double bond is sterically hindered. This suggests that O­(^3^P) interacts with adsorbed 1-hexene primarily through the sp^3^ carbons oriented away from the surface. As suggested by the quantum chemical calculations of binuclear cluster Al_2_O_3_-1-hexene, O­(^3^P) likely reacts through two pathways, as suggested in Scheme 1. First, oxidation is initiated with O­(^3^P) abstracting a hydrogen from the fourth or fifth carbon of 1-hexene, forming a secondary alkyl radical and hydroxyl radical (·OH). The alkyl radical can recombine with ·OH to form alcohol groups, which may undergo further oxidation to form ketones. While O­(^3^P) is the primary driver of oxidation due to its high reactivity, alkyl radicals can also react with excess molecular oxygen, generating unstable peroxyl radicals that rapidly disproportionate to yield an alcohol and a ketone. An alternative reaction pathway involves adsorbed 1-hexene molecules reacting with O­(^3^P) via the double bond. In this case, oxidation is initiated by the electrophilic addition of O­(^3^P), forming a biradical intermediate that leads to the production of adsorbed 2-hexenol, which undergoes further oxidation under the continuous flow of the nonthermal plasma. The data support these reaction pathways; as 2-hexenol is detected, ketones formed in the single-bonded carbons are also detected, and combined alcohols and ketones ultimately emerge as the primary products in 4,5-hexenedione-2-ol. The products detected via GC–MS are highlighted in Scheme 1.

The oxidation of adsorbed cyclohexane by the compound O­(^3^P) is outlined in Scheme 2. Quantum chemical calculations of the binuclear Al_2_O_3_-cyclohexane cluster indicate fewer symmetric constraints in adsorbed cyclohexane compared to 1-hexene. Consequently, a broader range of products is observed, each with yields below 15%. Similar to the oxidation of sp^3^ carbons in 1-hexene, the reaction begins with O­(^3^P) abstracting a hydrogen from cyclohexane, forming an alkyl radical and ·OH. The recombination of these two species results in the formation of cyclohexanol, which, like the initial oxidative products of adsorbed 1-hexene, can undergo further reactions to form diols and ketone groups. While disproportionation reactions can generate double bonds, they also lead to the formation of epoxide groups. Figures 4 and 5 show the initial formation and subsequent depletion of the OH vibrational band, supporting the mechanism proposed in Scheme 2. The GC–MS data also align with the products highlighted in Scheme 2, with ketones emerging as the principal products at the end of the reaction, while some OH functional groups remain present.

### 3.3 Comparative Kinetic Analysis

As indicated by eq 1, the oxidation reactions with the nonthermal oxygen plasma occur via a bimolecular process, through second-order rate kinetics, as described below

2 d[Organic]dt=−kJO(P3)θ=dθdt

where J_O(_^3^_P)_ is the molar flux of O­(^3^P) and θ represents the coverage of the two isomers of C_6_H_12_ investigated in this study on the α-Al_2_O_3_ surface, which is assumed to be monolayer or submonolayer in coverage. Since the molar flux is kept constant throughout the experiment, rearranging and integrating eq 2 through the reaction time, *t*, gives

dθθ=−kJO(P3)dt⇒∫0tdθθ=−kJO(P3)∫0tdt

3 ln⁡θtθ0=−kJO(P3)t

Since coverage is limited to a monolayer, the average molar absorptivity of adsorbed 1-hexene or cyclohexane can be assumed constant, allowing the use of Beer–Lambert’s law to estimate changes in surface concentration.^70^ In other words, when limited to a monolayer, the surface coverage is approximately proportional to the absorbance of C_6_H_12_ on the surface, θ_t_∝Abs. Therefore, eq 3 can be written in terms of absorbance

4 ln⁡AbsAbs0=−kJO(P3)t

As long as O­(^3^P) reacts with chemisorbed C_6_H_12_, eq 4 suggests a linear relation between $\ln\frac{Abs}{{Abs}_{0}}$ and the reaction time. The slope (*m*) of the corresponding plot is given by *m* = -*k*J_O(^3^P)_. To minimize interference from overlapping vibrational signals and considering that the rate-determining step in the surface-mediated oxidation of both 1-hexene and cyclohexane involves H-abstraction by O­(^3^P), the ν_CH_ spectral region provides the most reliable measure of absorbance for constructing the $\ln\frac{Abs}{{Abs}_{0}}$ vs time plot. Figure 7 shows the natural logarithm of the ratio of the integrated ν_CH_ absorbances for adsorbed 1-hexene and cyclohexane as they react with O­(^3^P).

**Figure 7:**
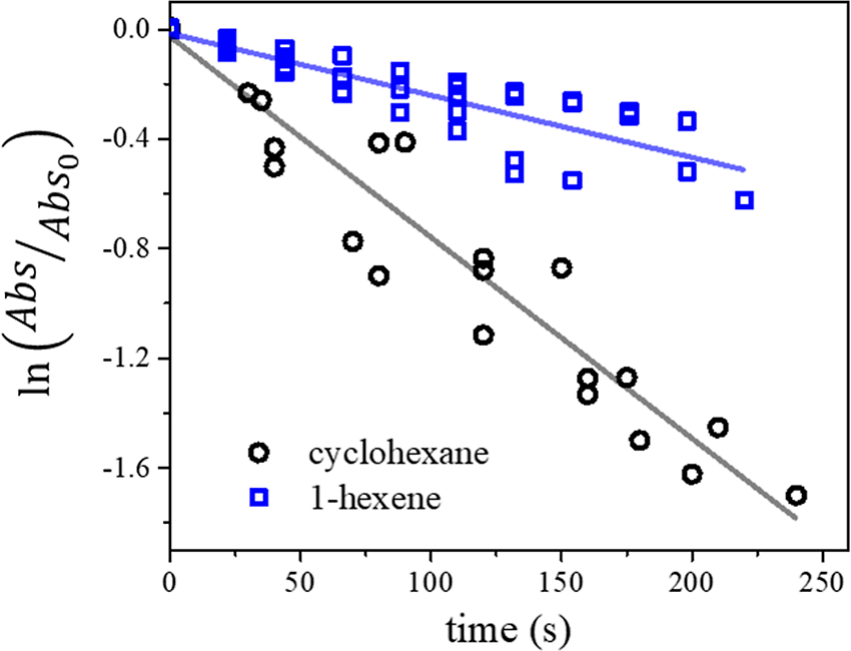
Natural logarithm of the relative decrease in the C–H absorbance intensity. Blue squares represent at least triplicate experiments of adsorbed 1-hexene; black circles represent at least triplicate experiments of adsorbed cyclohexane. The line represents the linear regression over the pseudo-first-order kinetics regime used to delimit the exponential decay of adsorbed hydrocarbons.

Given the experiments were conducted under constant oxygen flux, the ratio between the slopes for 1-hexene (*m*_h_) and cyclohexene (*m*_c_) is proportional to the ratio between kinetic rate constants

5 mhmc∝khJO(P3)kcJO(P3)=khkc

where *k*_*h*_ is the kinetic constant for the reaction between adsorbed 1-hexene and O­(^3^P), while *k*_c_ is the same but for adsorbed cyclohexane. Thus, using the slopes extracted from Figure 7, the ratio between *k*_h_ and *k*_c_, as described in eq 5, is 0.48 ± 0.07. This indicates that, while adsorbed on the surface of α-Al_2_O_3_, the cyclohexane kinetic constant for its reaction with O­(^3^P) is twice that of 1-hexene. Conversely, the ratio of the gas-phase reaction rate constants for 1-hexene and cyclohexane, as reported by Atkinson et al., indicates that the reaction of 1-hexene with O­(^3^P) is 37-times faster.^71^ Thus, the net effect of the interface reaction is a substantial decrease in the reaction rate constant of 1-hexene, *k*_h_, to a greater extent than the corresponding decrease for cyclohexane. We interpret this reversal in the kinetic constant ratio as a consequence of a dual surface effect on the adsorption of 1-hexene. First, the adsorption of 1-hexene onto α-Al_2_O_3_ is energetically favored when the double bond is interacting with the surface, as shown through B3LYP calculations (Figure 3A). Consequently, the O­(^3^P) plume encounters a monolayer, where the σ bonds at the saturated end of the molecule are more accessible than the π bonds. Second, while less significant than steric hindrance of the double bond, the chemisorption process withdraws electrons from the substrate, resulting in a surface-bound 1-hexene with a π-bond system that is less electron-dense than in the gas phase. This results in a slightly higher activation between adsorbed 1-hexene and the electrophilic O­(^3^P) compared to the gaseous reaction. The combination of the adsorption geometry shielding the π-bonds and the slight loss in electron density in the π-bonds of 1-hexene due to chemisorption ultimately leads to adsorbed 1-hexene to exhibit reactivity more analogous to an alkane than an alkene. In fact, the ratio of the kinetic constants of the reaction between gaseous hexane and cyclohexane with O­(^3^P) is 0.67, with the cyclohexane kinetic constant being about 50% higher than hexane (C_6_H_14_).^71^ This proportion is consistent with the observed ratio between *k*_c_ and *k*_h_ when C_6_H_12_ is adsorbed onto a surface, suggesting that the reaction of 1-hexene slows and becomes kinetically comparable to that of an alkane reaction. On the other hand, the symmetry of cyclohexane does not offer a significant change between the gaseous phase and adsorbed phase, with just a slight breaking from the D_3d_ point group, but largely preserving the chair configuration, as shown in Figure 3B. Thus, the overall surface effect in the reaction of O­(^3^P) with adsorbed cyclohexane is a decrease in rate, driven by the restricted degrees of freedom of chemisorbed cyclohexane compared to its gaseous state.

## 4 Conclusions

This work describes a system for the heterogeneous functionalization of volatile hydrocarbons using nonthermal plasmas. The system consists of a reaction chamber equipped with in situ vibrational spectroscopy for real-time analysis and optimized for studying the reactions of surface-bound volatile hydrocarbons with low-pressure fluxes of O­(^3^P) generated by nonthermal plasmas. The reactions are carried out on a monolayer coverage of chemisorbed hydrocarbons, which are then exposed to plasma-induced oxidation. Here, the reaction system was used to conduct the heterogeneous oxidation of two isomers of C_6_H_12_, 1-hexene and cyclohexane, using nonthermal oxygen plasma. Combined with theoretical geometry optimization and ex situ GC–MS analysis for product characterization, this system enabled a comparative kinetic study of the reaction and provided insight into surface effects.

The combination of in situ vibrational spectroscopy and ex situ GC–MS analysis reveals that the nonthermal plasma heterogeneous oxidation of chemisorbed 1-hexene and cyclohexane on α-Al_2_O_3_ produces surface-bound ketones and alcohols, along with some recombination and dimerization leading to higher-mass products. The adsorption process has a dual effect. First, it enables an Eley–Rideal-type reaction where O­(^3^P) reacts with surface-bound C_6_H_12_, leading to greater selectivity while preventing the hydrocarbon from partitioning into the plasma phase. Second, it restricts the mobility of adsorbed C_6_H_12_, introducing steric constraints that influence collisions between surface-bound molecules and the nonthermal plasma flux.^72,73^ The effect of mobility restrictions is most pronounced in the less symmetric of the two isomers studied, 1-hexene. In this case, the energetically favored orientation positions the double bond near the surface, exposing the sp^3^ carbons to the nonthermal plasma flux. As a result, the adsorbed molecule behaves partially as an alkane, leading to relatively slower reaction rates and the formation of oxidation products away from the double bond. Cyclohexane, being more symmetric, is less affected by the surface effect as the segment of the molecule near the surface is equivalent to that away from it. As a result, the reaction between adsorbed cyclohexane and O­(^3^P) is not as selective as the reaction in involving adsorbed 1-hexene. Ultimately, adsorbed cyclohexane is two times faster than 1-hexene, a significant change from the gas phase reactivity with O­(^3^P), where 1-hexene reacts 37 times faster than cyclohexane. These types of surface effects have the potential of affecting reactions with other free radicals.^74^

The nonthermal plasma oxidation of adsorbed hydrocarbons discussed in this work provides insights into a functionalization process that requires low pressures without gas-phase partitioning of the volatile substrates. The results shown in this work provide further insight on the surface effects of heterogeneous nonthermal plasma reactions and how the adsorption process influences the pathways for these interface reactions. While providing an alternative for the use of byproducts of petroleum refinement,^9,22–24,68,74^ the technique presented in this work also offers a potential for the study of processes involving free radicals, such as atmospheric oxidation,^36,61^ ozonolysis,^13–15^ and the in situ study of the direct functionalization of surfaces involving plasma systems.^75–79^

## Supplementary Material



## Notes

Calculation of the quenching lifetime of O­(^1^D) by O_2_ in the nonthermal plasma (PDF)
