# Supplementary material for: Probing O(3P) Reactivity with Chemisorbed Hydrocarbons: Insights from Experiment and Theory
Source: J Phys Chem A. 2025 Jun 3;129(27):6094–106. doi: 10.1021/acs.jpca.5c01709 (PMC12257514; doi:10.1021/acs.jpca.5c01709)
Supplement: Supplementary file 1 [file jp5c01709_si_001.pdf]

Supplementary Information

Probing $O(^3P)$ Reactivity with Chemisorbed Hydrocarbons: Insights from Experiment and Theory

Claudia Bennett-Caso, Angelina L. Leonardi, Rachel Hambuchen, Aida Castelblanco, Jack Spagnoletti, Cecily Szady, Natasha Wozniak, Juan G. Navea*

Chemistry Department, Skidmore College, Saratoga Springs, NY, 12866-1632, USA

* Corresponding author: Juan G. Navea, jnavea@skidmore.edu

The pseudo-first-order rate constant for the quenching of $O(^1D)$ by O_2 in the plasma was calculated for a system at 140 mTorr, with the reaction chamber volume of 100.45 cm^3 and a temperature of 298 K. The quenching of $O(^1D)$ is primarily driven by O_2, the main component in the plasma, following the reaction:[1,2]

$$O(^1D) + O_2 \longrightarrow O(^3P) + O_2 \quad (S.1)$$

with a bimolecular rate constant ($k_{bimolecular}$) of 4×10^{-11} cm^3 $molecules^{-1}$ s^{-1}.[1,2]

The number density of O_2 molecules is calculated using the ideal gas law, where the number of moles of O_2 is

$$n = \frac{(0.140\ torr)(100.45\ cm^3)}{(62365.6\ \frac{torr \cdot cm^3}{mol \cdot K})(298\ K)} = 7.566 \times 10^{-7} mol$$

And the number density is given by:

$$N_{O_2} = \frac{n \times N_A}{V} \quad (S.2)$$

Where N_A is Avogadro's number. Thus,

$$N_{O_2} = \frac{(7.566 \times 10^{-7} mol)(6.022 \times 10^{23} molecules\ mol^{-1})}{100.45\ cm^3} = 4.54 \times 10^{15} \frac{molecules}{cm^3}$$

The pseudo first order constant,

$$k_{pseudo} = k_{bimolecular} \times N_{O_2} \quad (S.3)$$

$$k_{pseudo} = (4 \times 10^{-11}\ cm^3\ molecules^{-1} s^{-1})(4.54 \times 10^{15}\ molecules\ cm^{-3})$$

$$k_{pseudo} = 1.816 \times 10^5 s^{-1}$$

With the lifetime τ of $O(^1D)$ due to quenching by O_2 is the inverse of the pseudo first-order rate constant, τ = 5.5 μs.

References

1. Atkinson, R., Baulch, D. L., Cox, R. A., Crowley, J. N., Hampson, R. F., Hynes, R. G., Jenkin, M. E., Rossi, M. J., Troe, J. Evaluated kinetic and photochemical data for atmospheric chemistry: Volume I – gas phase reactions of O_x, HO_x, NO_x and SO_x species. *Atmos. Chem. Phys.*, 2004, 4, 1461–1738.
2. Dunlea, E. J., Ravishankara, A. R. Measurement of the rate coefficient for the reaction of $O(^1D)$ with H_2O and re-evaluation of the atmospheric OH production rate. *Phys. Chem. Chem. Phys.* 2004, 6, 3333–3340.
